# Impact of mGFR Versus eGFR on the Pharmacokinetics of a Renally Excreted Model Drug in Older Medical Patients: A Proof-of-Concept Trial

**DOI:** 10.1016/j.ekir.2026.107020

**Published:** 2026-08-17

**Authors:** Ida K. Storgaard, Morten B. Houlind, Louise WS. Christensen, Rikke L. Nielsen, Aino L. Andersen, Olivia Bornæs, Helle G. Juul-Larsen, Johnny K.H. Dang, Baker N. Jawad, Izzet Altintas, Juliette Tavenier, Esteban Porrini, Esben Iversen, Morten Damgaard, Ove Andersen, Mads Hornum, Trine M. Lund

**Affiliations:** 1 Department of Clinical Research, Copenhagen University Hospital, Amager and Hvidovre, Hvidovre, Copenhagen, Denmark; 2 Department of Drug Design and Pharmacology, Faculty of Health and Medical Sciences, University of Copenhagen, Copenhagen, Denmark; 3 Department of Nephrology and Endocrinology, Copenhagen University Hospital Rigshospitalet, Copenhagen, Denmark; 4 Department of Clinical Medicine, Faculty of Health and Medical Sciences, University of Copenhagen, Copenhagen, Denmark; 5 Department of Geriatric Medicine and Palliative Care, Amager and Hvidovre Hospital, Hvidovre, Copenhagen, Denmark; 6 Department of Emergency Medicine, Copenhagen University Hospital, Amager and Hvidovre, Hvidovre, Denmark; 7 Laboratory of Renal Function (LFR), Faculty of Medicine, University of La Laguna, La Laguna, Spain; 8 Department of Clinical Physiology and Nuclear Medicine, Centre for Functional and Diagnostic Imaging and Research, Copenhagen University Hospital Amager and Hvidovre, Hvidovre, Copenhagen, Denmark

**Keywords:** drug dosing, geriatrics, gentamicin, glomerular filtration rate, malnutrition, population pharmacokinetic modeling

## Abstract

**Introduction:**

Accurate assessment of kidney function is essential for dosing renally eliminated drugs, such as gentamicin. However, creatinine-based estimated glomerular filtration rate (eGFR) equations may be inaccurate in older adults because creatinine concentrations are influenced by nonrenal factors, including reduced muscle mass and frailty. This study investigated whether measured GFR (mGFR) and 10 different eGFR equations improve the modeling of gentamicin renal clearance, used as a model compound for renally eliminated drugs, when included as covariates in a population pharmacokinetic (popPK) model of older medical patients with poor appetite.

**Methods:**

This prospective diagnostic accuracy and population pharmacokinetic study evaluated mGFR determined by 99mTc-DTPA plasma clearance and 10 eGFR equations on the basis of plasma creatinine, cystatin C, β2-microglobulin, and/or β-trace protein. Poor appetite was assessed using the Simplified Nutritional Appetite Questionnaire (SNAQ). Plasma gentamicin concentrations were measured over 22 hours after a single i.v. dose of 5 mg/kg. Gentamicin concentration–time profiles were analyzed using nonlinear mixed-effects modeling. Improvement in model performance was assessed by changes in objective function value (OFV) and interindividual variability (IIV) in clearance.

**Results:**

A total of 52 older medical patients with poor appetite (median age 79.5 [interquartile range, {IQR} 73.0–84.3] years, 56% female, median SNAQ score 12 [IQR 11–13]) were included. mGFR expressed in ml/min provided the greatest improvement in model fit when included as a covariate for gentamicin clearance (−ΔOFV = 115.00). Among eGFR equations, combined creatinine–cystatin C equations expressed in ml/min yielded the best performance (−ΔOFV = 72.58–77.11), whereas creatinine-only equations showed the poorest improvement (−ΔOFV < 63.69). Overall, absolute GFR estimates performed better than body surface area-indexed estimates.

**Conclusions:**

Creatinine-only eGFR equations may be suboptimal proxies for gentamicin clearance in this patient population. If measuring GFR is unfeasible, estimating GFR on the basis of a combination of creatinine and cystatin C is the best alternative.

## Graphical abstract

**Figure:**
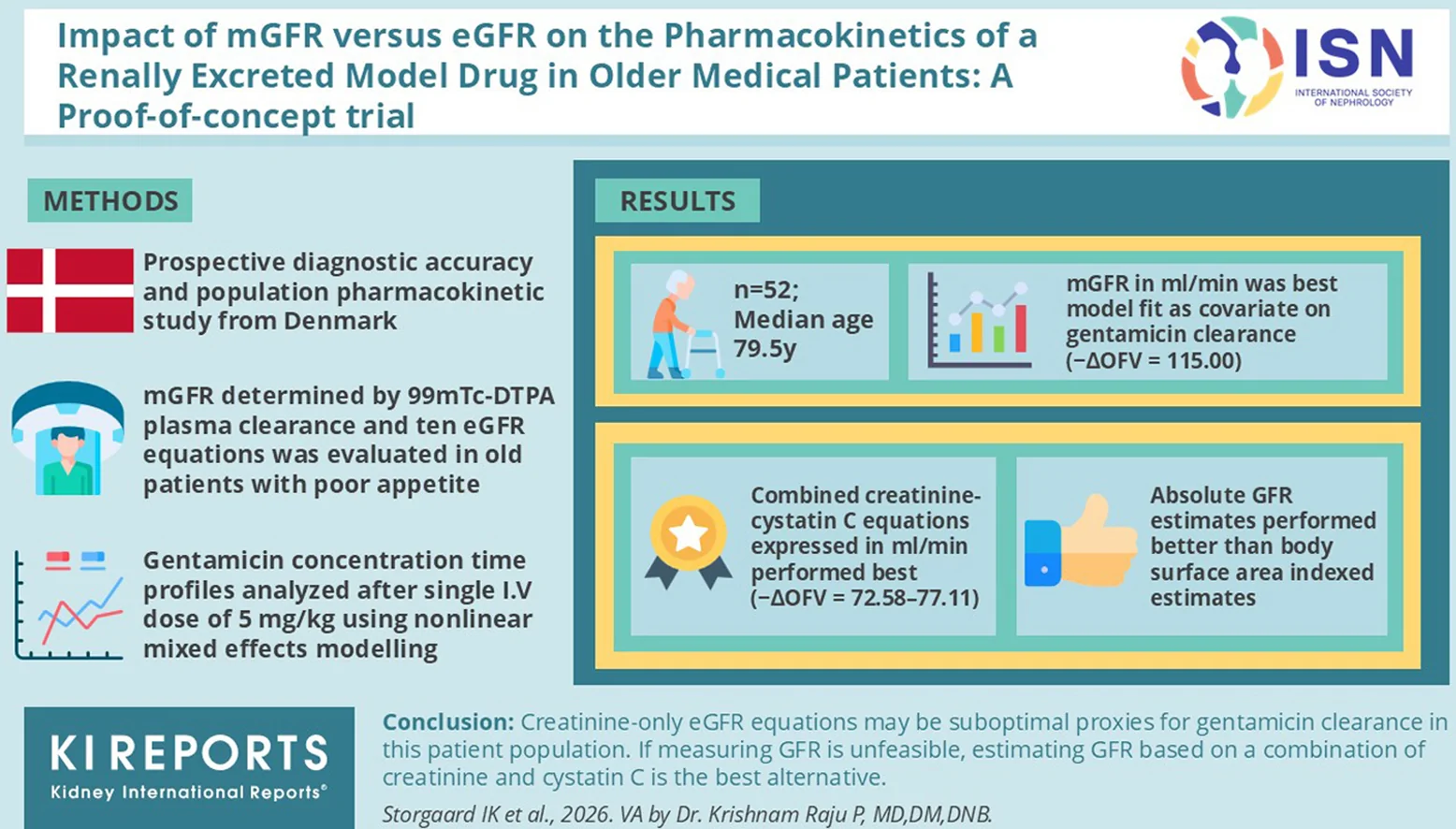

The global population of older adults aged ≥ 65 years is projected to double from 703 million in 2019 to 1.5 billion in 2050.^1^ This poses major challenges for health care systems, particularly emergency departments, where older adults account for around 40% of acute admissions.^2^, ^3^, ^4^, ^5^ Age-related physiological changes, multimorbidity, malnutrition, and widespread polypharmacy substantially increase the risk of inappropriate medication dosing.^6^, ^7^, ^8^ Acutely admitted older adults are also characterized by poor appetite, an important risk factor of malnutrition, which is strongly associated with weight loss, muscle wasting, altered creatinine levels, and poor clinical outcomes.^9^, ^10^, ^11^, ^12^, ^13^, ^14^, ^15^, ^16^, ^17^

Accurate assessment of GFR is crucial for diagnosing and staging chronic kidney disease (CKD) as well as for dosing medications that require dosing according to kidney function (hereafter, renal-risk medications),^18^^,^^19^ which constitute for approximately 40% of all prescriptions.^20^ In clinical practice, dosing of renal-risk medications is typically performed using estimated creatinine clearance (eCrCL) or eGFR based on creatinine (eGFR_CR_).^18^^,^^21^ However, creatinine levels are strongly influenced by nonGFR factors, such as sex, age, muscle mass, and nutritional status,^22^, ^23^, ^24^ often leading to errors exceeding 30% compared with true GFR.^23^^,^^25^ These errors are magnified in acutely admitted older adults, of whom one-thirds to two-thirds have malnutrition,^12^, ^13^, ^14^, ^15^, ^16^, ^17^ and pose a substantial risk of inappropriate drug dosing that can lead to toxicity, adverse drug reactions, acute kidney injury (AKI), prolonged hospitalization, and other adverse health outcomes.^18^^,^^26^, ^27^, ^28^, ^29^

Cystatin C, β-2 microglobulin, and β-trace protein are alternative filtration markers that are less dependent on sex, age, muscle mass, and nutritional status compared with creatinine.^22^^,^^23^^,^^30^ However, they may be influenced by other nonrenal factors, such as chronic inflammation, thyroid function, obesity, smoking, and corticosteroid use.^22^^,^^23^ Current evidence indicates that GFR estimates based on cystatin C alone (eGFR_CYSC_) do not outperform eGFR_CR_, but that GFR estimates combining creatinine and cystatin C (eGFR_COMB_) outperform both eGFR_CYSC_ and eGFR_CR_ across different age groups and patient populations.^31^, ^32^, ^33^ We have previously shown that both eGFR_COMB_ and a GFR estimate derived from a panel of creatinine, cystatin C, β-2 microglobulin, and β-trace protein (eGFR_PANEL_) outperform eGFR_CR_ in older medical patients, and that this difference in performance is even more pronounced among patients with malnutrition.^34^ The most recent 2024 Kidney Disease: Improving Global Outcomes (KDIGO) recommendations suggest measuring cystatin C in clinical situations where eGFR_CR_ is considered unreliable.^18^ Alternatively, GFR can be measured directly using an exogenous tracer, but mGFR is resource-intensive and time-consuming compared with eGFR.^18^^,^^35^

Despite considerable progress in developing and validating novel filtration markers for diagnostic purposes, evidence regarding their implications for medication dosing—particularly in older adults—remains limited. Only a few studies have evaluated eGFR_COMB_ in pharmacokinetic models that form the basis for dosing recommendations.^19^^,^^36^, ^37^, ^38^ To date, no studies have assessed eGFR_PANEL_ in this context. Furthermore, few pharmacokinetic studies incorporate mGFR, making it difficult to evaluate the potential for improvement by switching between eGFR equations. Therefore, there is an unmet need for more precise and individualized dosing of renal-risk medications in older medical patients with poor appetite. To address this, we conducted a proof-of-concept study in older medical patients with poor appetite to compare mGFR and 10 different eGFR equations by applying popPK modeling to assess the extent to which each method of GFR assessment improves model fit when included as a covariate for renal clearance, using gentamicin as a model drug.

## Methods

### Study Design and Setting

This study reports findings from a protocolized prospective, investigator-initiated, single-center clinical trial investigating methods for assessing GFR in older medical patients with poor appetite. A subset of patients recruited for this study (substudy II) also participated in a separate substudy (substudy I), the results of which have been reported previously.^39^^,^^40^ Participation in substudy I did not influence the outcomes of the present study. The original trial was conducted at Copenhagen University Hospital, Hvidovre, Denmark (hereafter, Hvidovre Hospital) between February 2022 and January 2025. Details of the original trial have been reported previously in the trial protocol.^41^ The original trial was carried out in accordance with the Declaration of Helsinki and approved by the ethics committee of the Capital Region of Denmark (H-21044231), the Danish Medicines Agency (EudraCT no. 2021-002318-15), and the Danish Data Protection Agency (Journal no. P-2021-744), registered at Clinicaltrials.gov (NCT05503147), and monitored by the Good Clinical Practice unit of the University of Copenhagen according to the ICH E6 (R2) Guideline for Good Clinical Practice. Informed written consent was obtained from all patients.

### Selection Criteria

Patient inclusion took place consecutively at the Emergency Department, Hvidovre Hospital. As protocolized, patients could participate in substudy I,^41^ in the present study (substudy II), or in both substudies (Supplementary Figure S1 for details). Participation in trial days took place after a recovery period of at least 2 weeks after acute admission. For patients participating in both substudies, a minimum of 14 days separated the trial days of each study. Once recruitment for substudy I was completed, patients were only eligible for inclusion in substudy II.

The selection criteria for substudy II are described in detail in the Supplementary Table S1. Key criteria were age ≥ 65 years and a SNAQ score ≤ 14 points. The SNAQ is widely used in clinical practice to identify patients with malnutrition as well as those at risk of developing it.^42^, ^43^, ^44^ Patients with eGFR ≤ 15 ml/min per 1.73 m^2^ for >3 months or dialysis treatment were excluded.

Among those who had completed substudy II, patients were subsequently excluded from the present analysis if they had a body mass index (BMI) > 26 kg/m^2^ on the trial day. This was done to isolate patients with BMI within or below the normal range. Patients were also excluded if they had received immunosuppressants on the trial day or within the preceding 7 days, or if they presented with AKI on the trial day. AKI was defined as an increase in serum creatinine of ≥ 0.3 mg/dl or ≥ 50% from baseline.^45^

Data, such as patient characteristics (e.g., height and body weight) and exact time points for blood sampling, were collected in REDCap (Research Electronic Data Capture, Vanderbilt University, Nashville, USA). Additional medical history (e.g., age, chronic conditions, smoking status), social history (e.g., living conditions, use of outdoor mobility aids, municipal home-care support, falls within the previous year), list of prescribed medications, and 1-year mortality were obtained from electronic patient records. Nutritional status was evaluated using the Nutritional Risk Screening 2002, where a score of 3 or higher indicated nutritional risk.^46^ Sarcopenia was identified according to the European Working Group on Sarcopenia in Older People 2 recommendations.^47^^,^^48^ Low muscle mass was defined as appendicular skeletal muscle mass < 20 kg in men or < 15 kg in women, or appendicular skeletal muscle mass/height^2^ < 7.0 kg/m^2^ in men or < 5.5 kg/m^2^ in women.

### Trial Day

On the morning of the trial day, patients had a peripheral venous catheter applied to each arm (1 for GFR measurement and 1 for gentamicin sampling). Baseline blood samples were drawn before administration of a dose of 5 mg/kg gentamicin (Hexamycin 40 mg/ml, Sandoz A/S). The external catheter port was replaced after administration to avoid contamination of blood samples. Blood samples for analysis of gentamicin plasma concentrations were collected in ethylenediamine tetraacetic acid tubes at 10, 15, 30, 180, 240, 330, 345, and 360 minutes after dosing. The sampling time points had previously been determined on the basis of a model-informed approach using the PopED package in R^49^^,^^50^ and a popPK model of gentamicin from the literature.^51^ Body composition parameters (e.g., appendicular muscle mass and fat mass) were measured by dual-energy X-ray absorptiometry scan (GE Lunar Prodigy Primo, GE Healthcare Technologies, Madison, WI).^52^ A follow-up blood sample was collected after approximately 22 hours in a location convenient to the patient (e.g., their home). Samples were centrifuged (2200 *g*, 15 minutes) and plasma was isolated and stored at −80 °C until analysis.

GFR was measured using 99m-technesium-diethylenetriaminepentaacetic acid (99m-Tc-DTPA) plasma clearance according to standard methods at the Department of Clinical Physiology and Nuclear Medicine, Centre for Functional and Diagnostic Imaging and Research, Hvidovre Hospital.^53^^,^^54^ In short, 20 MBq of 99m-Tc-DTPA was injected through i.v., and venous blood samples were collected at 180, 200, 220, and 240 minutes after injection for patients with eGFR > 30 ml/min per 1.73 m^2^, or 180, 210, 240, 270, and 300 minutes after injection for patients with eGFR ≤ 30 ml/min per 1.73 m^2^ as previously described.^34^

### Assays

Gentamicin plasma concentrations were determined using a homogenous particle-enhanced turbidimetric immunoassay (the QMS Gentamicin assay^55^). If samples had concentrations > 10.0 μg/ml, they were manually diluted and reanalyzed. The lower limit of quantification was 0.3 μg/ml and the coefficient of variance was 2.8%–3.9%.^55^ Plasma levels of creatinine, cystatin C, β-2 microglobulin, β-trace protein, albumin, and soluble urokinase plasminogen activator receptor were determined using standard methods as previously reported.^34^ Plasma levels of growth differentiation factor 15 were analyzed using a Simple Plex Human Cartridge (SPCKB-PS-000269, ProteinSimple, San Jose, CA) on the Ella Platform (ProteinSimple). All samples were analyzed on the same cartridge along with commercially available high and low control samples, which were measured within 10% of the expected concentration. The average coefficient of variance of all samples was 1.8%.

### GFR Estimation

A total of 10 equations were used to estimate kidney function—1 for eCrCL (Cockcroft-Gault), 3 for eGFR_CR_ (CKD-Epidemiology Collaboration [CKD-EPI] 2009, CKD-EPI 2021, and European Kidney Function Consortium [EKFC]), 2 for eGFR_CYSC_ (CKD-EPI 2012 and EKFC), 3 for eGFR_COMB_ (CKD-EPI 2012, CKD-EPI 2021, and EKFC), and 1 for eGFR_PANEL_ (CKD-EPI 2021) (Supplementary Table S2 for details). All values were calculated in both absolute units (ml/min) and body surface area (BSA)-indexed units (ml/min per 1.73 m^2^), where BSA was calculated with the Du Bois method.^56^^,^^57^

### Metrics for GFR Performance

The primary metric for comparing mGFR, eCrCL, and eGFR was the change in the maximum likelihood estimation OFV (equal to the –2 log likelihood), which has approximately a χ^2^ distribution. When comparing nested models, a lower OFV indicates a better model. A significant improvement in model fit corresponding to a *P*-value of 0.05 with 1 degree of freedom would result in a decrease in OFV of 3.84. Secondary metrics included reduction in IIV on clearance for covariate models compared with the base model, and simulation-based visualization of covariate impact on the area under the gentamicin plasma concentration-time curve (AUC) for assessment of clinical relevance.

### Population Pharmacokinetic Modeling

Data were handled in R (version 4.3.2)^58^ and nonlinear mixed-effects modeling of gentamicin plasma concentrations over time was conducted using NONMEM (version 7.5, ICON plc, Dublin, Ireland) with Perl (version 5.34.0, ActiveState, Vancouver, BC, Canada) and Perl-speaks-NONMEM (version 5.2.6), using Pirana (version 24.9.2, Certara, Princeton, NJ) as a graphical user interface. Data below the limit of quantification were treated as missing (M1 method^59^).

Data were visually inspected prior to modeling to identify trends that could influence model development and to detect potential outliers, defined as sudden, single point drops or spikes in which the observation deviated >30% from the mean of the 2 surrounding values.

Models were run using the FOCE algorithm with interaction. One-, 2- and 3-compartment models were fitted to the data, and the best model was chosen. Different combinations of implementing IIV on the structural parameters were tested, along with additive, proportional, and combined additive and proportional error models to describe residual unexplained variability. The best structural and stochastic model was considered the base model.

Assessment criteria considered during model development were OFV, precision of parameter estimates, goodness-of-fit plots, overall stability of the model, and shrinkage of stochastic parameters, which should be below 25% for post hoc (individual) parameter estimates to be considered reliable as basis for covariate analysis. Robustness and validity of the base model, intermediate model, and final model were assessed by visual predictive checks and nonparametric bootstrap analysis (*n* = 1000 for both). 95% CIs for the model parameters were also obtained from the bootstrap analysis.

### Covariate Analysis

Covariate analysis was divided into 2 phases. First, mGFR and the 10 eGFR equations (in both absolute and BSA-indexed units) were individually added as covariates on gentamicin clearance in the base model to compare the extent to which they improved model fit. Improvement in model fit was evaluated by changes in OFV, IIV, and simulation-based assessment of covariate effects on exposure. Second, the best indicator of kidney function was included in the model, and the stepwise method for evaluating the remaining potential covariates was applied to obtain the final model. The criteria for a decrease in OFV were at least 3.84 (*P* < 0.05) for forward selection and 6.63 (*P* < 0.01) for backward elimination.

Categorical covariates (e.g., sex) were implemented linearly with the most common category for the typical value and an extra parameter added for each additional state. Continuous covariates (e.g., kidney function, body composition parameters, laboratory values) were implemented using a power relation as exemplified in equation (1) for clearance.

(1) $${\text{CL}}_{i}=\theta_{\text{TVCL}}\cdot \exp\left(\eta_{\text{CL}i}\right)\cdot {\left({\text{COV}}_{i}/{\text{COV}}_{\text{ref}}\right)}^{\theta_{\text{COV}}}$$

Where CL_*i*_ is the individual clearance, $\theta_{\text{TVCL}}$ is the typical or population value for clearance, $\eta_{\text{CL}i}$ is the IIV for clearance, COV_*i*_ is the covariate value for the individual, COV_ref_ is the reference value (in this case, the median for the study population), and $\theta_{\text{COV}}$ is the covariate effect.

### Simulations

For a comparative assessment of the clinical relevance of mGFR and eGFR equations as covariates on clearance, we used the 11 models from the first phase of the covariate analysis to simulate gentamicin exposure (AUC) for subjects with GFR or CrCL of 90, 60, and 30 ml/min. The mean exposure for a subject at the lower limit of normal kidney function (90 ml/min) was used as the reference in each case. Simulations were performed using the *nlmixr2* (integrating *rxode2*) package in R.^60^ Plasma concentrations were simulated for every 0.1 hour over 1 week after a dose of 350 mg (5 mg/kg for a 70-kg subject, *n* = 1,000 for each subject and method) and AUC was calculated using the trapezoidal method. Median and 90% CI were then presented for each kidney function category relative to the reference in a forest plot.

### Performance Statistics

We calculated additional performance statistics of the various eGFR equations compared with mGFR, that is, bias, relative bias, root mean squared error, and the percentage of values within ±30% and ±20% of mGFR (P30 and P20, respectively), using R (version 4.3.2).^58^ 95% CIs were obtained using a nonparametric bootstrap analysis (*n* = 10,000).

## Results

### Demographic and Patient Characteristics

A flowchart of patient inclusion is presented in Supplementary Figure S1. In total, 4465 patients were screened, and 676 were approached for recruitment. Of these, 60 patients agreed to participate and completed the study. Of the 60 patients, 8 patients were ultimately excluded from the analysis because of BMI > 26 kg/m^2^, use of immunosuppressants, or AKI, resulting in a final cohort of 52 participants. Detailed reasons for exclusion and dropout are presented in Supplementary Tables S3 and S4.

Among the 52 patients included in this analysis, 29 (56%) were female, the median age was 79.5 years, the median mGFR was 55 ml/min, the median BMI was 22.6 kg/m^2^, the median SNAQ score was 12, the median plasma growth differentiation factor 15 was 2265 pg/ml, the median number of long-term medications was 7.5, and 75% of patients were exposed to polypharmacy (≥ 5 regular medications), whereas 54% had a history of falls within the past year. In total, 38 participants (75%) were at risk of malnutrition, 33 (69%) had low muscle mass, and 1-year mortality was 12% (Table 1). Hospital admissions were most frequently attributed to infectious diseases (*n* = 11) and pulmonary conditions (*n* = 11), followed by cardiovascular conditions (*n* = 10) and falls (*n* = 10), other causes (*n* = 8), and pain-related conditions (*n* = 4).

**Table 1:** Summary of patient demographics (total number of patients *N* = 52)

Characteristic	*n* (%) or median (IQR) [min–max]	
Sex		
Female	29 (56%)	
Male	23 (44%)	
Age (y)	79.5 (73.0, 84.3)	[67–96]
SNAQ score	12 (10, 13)	[5–14]
NRS-2002 ≥ 3^b^	38 (75%)	
Body weight (kg)	63 (50, 70)	[35–89]
BMI (kg/m^2^)	22.6 (20.1, 24.1)	[13.7–25.8]
BSA (m^2^)	1.69 (1.48, 1.84)	[1.29–2.16]
ASM (kg)^a^	15.6 (12.9, 18.0)	[7.7–25]
Appendicular skeletal fat mass (kg)^a^	8.01 (6.19, 9.50)	[3.86-14.2]
ASM below EWGSOP2 cut-off^a^	33 (69%)	
ASM/height^2^ below EWGSOP2 cut-off^a^	35 (73%)	
Smoking status		
Never	16 (31%)	
Former	25 (48%)	
Current	11 (21%)	
Living alone	31 (60%)	
Municipal home care services	26 (50%)	
History of falls within the past year	28 (54%)	
Use of mobility aids outdoors		
None	17 (33%)	
Cane	10 (19%)	
Walker	22 (42 %)	
Wheelchair	3 (6%)	
Blood markers		
Albumin (g/l)	34 (32, 37)	[17–41]
TSH mIU/L	1.60 (1.10–2.50)	[0.1–150]
CRP, mg/l	4.60 (1.70–9.21)	[0.3–143]
IL-6, pg/ml	6.91 (4.43–12.604)	[3.5–60.1]
suPAR (ng/ml)	4.93 (3.85, 5.68)	[2.09–24.47]
GDF-15 (pg/ml)	2,265 (1,730, 3,206)	[973–11,469]
Medication		
Long-term medications	7.5 (4.75, 10.0)	[0–16]
“As needed” medications	1.0 (1.0, 2.25)	[0–7]
Chronic conditions		
Diabetes	7 (13%)	
Hypertension	30 (58%)	
Cardiovascular disease	30 (58%)	
Cerebrovascular disease	12 (23%)	
Kidney disease	6 (12%)	
Pulmonary disease	32 (62%)	
Osteoporosis	16 (31%)	
Kidney function biomarkers		
Creatinine (mg/dl)	0.88 (0.66, 1.08)	[0.44–3.50]
Cystatin C (mg/l)	1.30 (1.00, 1.60)	[0.77–3.80]
β-2 microglobulin (mg/l)	2.87 (2.38, 3.86)	[1.55–19.20]
β-trace protein (mg/l)	0.76 (0.65, 0.96)	[0.37–3.44]
Measured kidney function^c^	ml/min	ml/min per 1.73 m^2^
mGFR	55 (44, 71) [13–109]	59 (47, 70) [13–121]
Estimated kidney function^c^	ml/min	ml/min per 1.73 m^2^
eCrCL (Cockcroft-Gault)	53 (39, 70) [12–111]	51 (43, 72) [13–103]
eGFR_CR_ (CKD-EPI 2009)	67 (57, 82) [14–105]	70 (59, 87) [15–101]
eGFR_CR_ (CKD-EPI 2021)	72 (61, 87) [16–110]	75 (63, 92) [16–103]
eGFR_CR_ (EKFC)	59 (51, 74) [14–94]	64 (53, 76) [14–91]
eGFR_CYSC_ (CKD-EPI 2012)	48 (34, 64) [11–101]	51 (36, 67) [12–94]
eGFR_CYSC_ (EKFC)	50 (36, 66) [15–88]	53 (40, 67) [16–82]
eGFR_COMB_ (CKD-EPI 2012)	57 (45, 74) [12–103]	61 (46, 74) [13–103]
eGFR_COMB_ (CKD-EPI 2021)	61 (47, 78) [13–110]	65 (48, 79) [14–110]
eGFR_COMB_ (EKFC)	55 (45, 70) [14–89]	59 (47, 69) [15–87]
eGFR_PANEL_ (CKD-EPI 2021)	58 (45, 78) [13–105]	63 (47, 75) [14–100]

ASM, appendicular skeletal muscle mass; BMI, body mass index; BSA, body surface area; CKD–EPI, Chronic Kidney Disease Epidemiology Collaboration; COMB, combined creatinine and cystatin C; CR, creatinine; CRP, C-reactive protein; CYSC, cystatin C; eCrCL, estimated creatinine clearance; eGFR, estimated glomerular filtration rate; EKFC, European Kidney Function Consortium; EWGSOP2, European Working Group on Sarcopenia in Older People 2; GDF–15, growth differentiation factor 15; IL-6, interleukin-6; IQR, interquartile range; mGFR, measured glomerular filtration rate; n, number of patients; NRS-2002, Nutritional Risk Score-2002; PANEL, filtration marker panel (creatinine, cystatin C, β-2 microglobulin, β-trace protein); SNAQ, short nutritional assessment questionnaire; suPAR, soluble urokinase plasminogen activator receptor; TSH, thyroid-stimulating hormone.

a *n* = 48.

b *n* = 51.

c The Du Bois formula was used to calculate BSA for conversion between BSA-indexed (ml/min per 1.73 m^2^) and absolute (ml/min) units.

**Figure 1:**
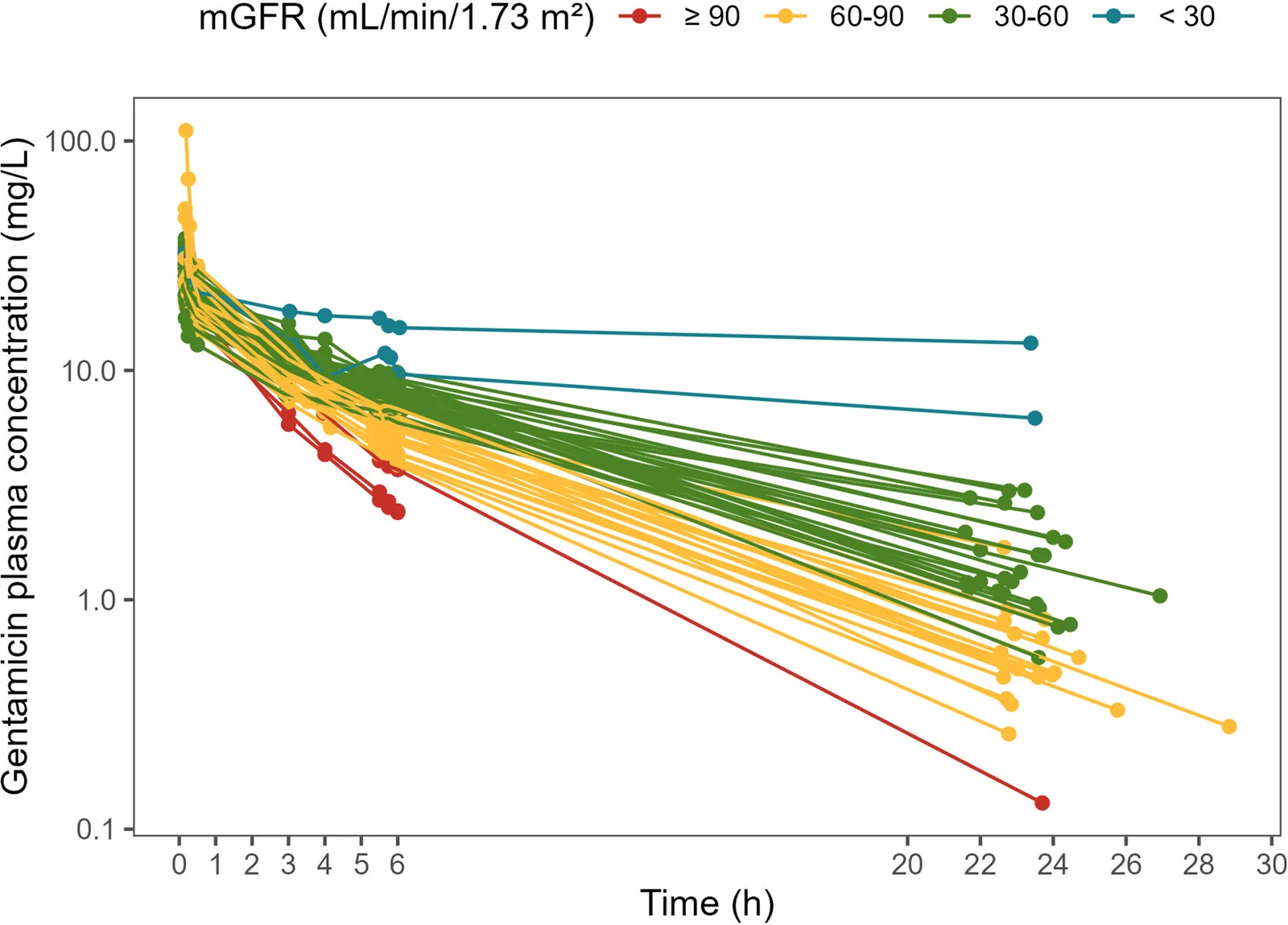
Semilogarithmic plot of gentamicin plasma concentrations over time. Colors indicate mGFR. mGFR, measured glomerular filtration rate.

In total, 465 blood samples were collected for analysis of gentamicin levels. As 2 outliers were identified and 3 observations (0.6%) were below the limit of quantification, a final total of 460 observations were used for popPK modeling. As seen in Figure 1, gentamicin clearance appeared to increase with higher mGFR.

### Base Model

A 2-compartment model provided a significant improvement in fit (ΔOFV = –445.51) compared with a 1-compartment model, whereas adding a third compartment did not improve model fit. The best model had IIV on CL, V1, Q, and V2 and a combined proportional and additive error model. The structure of the base model is presented in Supplementary Figure S2. Model-estimated pharmacokinetic parameters are shown in Table 2, and bootstrap results are shown in Supplementary Table S5. Goodness-of-fit plots and visual predictive checks are shown in Supplementary Figures S3–S5.

**Table 2:** Parameter estimates for the gentamicin base and final models

Parameter	Description	Base model		Final model	
		Estimate (RSE)	IIV, CV% (RSE) [Shrinkage]	Estimate (RSE)	IIV, CV% (RSE) [Shrinkage]
CL	Elimination clearance (L h^–1^)	2.27 (6.3%)	46.4% (22.5%) [1%]	2.31 (2.0%)	12.3 (19.0%) [10%]
Q	Intercompartmental clearance (L h^–1^)	7.55 (17.2%)	55.6% (18.8%) [24%]	8.05 (16.5%)	38.5% (43.1%) [37%]
V1	Distribution volume of the central compartment (L)	8.95 (6.7%)	60.6% (30.7%) [0%]	9.69 (4.8%)	44.8% (35.3%) [2%]
V2	Distribution volume of the peripheral compartment (L)	7.62 (7.8%)	27.3% (21.1%) [21%]	7.70 (7.6%)	24.7% (23.1%) [23%]
θ_CL-mGFR_	Covariate effect of mGFR on clearance (power function)	–	–	1.06 (7.9%)	–
θ_V1-WT_	Covariate effect of body weight on V1 (power function)	–	–	1.39 (38%)	–
RUV_CV_	Proportional residual error	0.0806 (9.7%)	–	0.0847 (8.5%)	–
RUV_SD_	Additive residual error (mg L^–1^)	0.215 (18.7%)	–	0.238 (18.8%)	–

CV, coefficient of variance; IIV, interindividual variability; RSE, relative standard error.

### Comparative Assessment of mGFR and eGFR Equations

Generally, GFR values expressed in absolute units (ml/min) performed better than values expressed in BSA-indexed units (ml/min per 1.73 m^2^) (Table 3). The covariate associated with the greatest improvement in model fit for gentamicin data was mGFR expressed in ml/min (ΔOFV = −115.0, decrease in IIV_CL_ from 46.4% to 13.8%). Among eGFR equations, eGFR_COMB_ and eGFR_PANEL_ were associated with greater improvements than eCrCL, eGFR_CR_ and eGFR_CYSC_. Overall, eGFR_CR_ was associated with the smallest improvement in model fit, and eCrCL expressed in ml/min was significantly worse than the other equations expressed in ml/min.

**Table 3:** OFV of models with different measures and estimates of kidney function as covariates on gentamicin clearance compared with the base model (OFV = 879.234, IIV_CL_ = 46.4%) (all covariates shown here were implemented with power functions)

Covariate	Unit	OFV	–ΔOFV	Covariate effect	IIV_CL_, CV%
mGFR	ml/min	764.236	114.998	1.06	13.8
mGFR	ml/min per 1.73 m^2^	797.813	81.421	1.05	19.7
eGFR_COMB_ (CKD-EPI 2021)	ml/min	802.120	77.114	1.01	20.2
eGFR_COMB_ (CKD-EPI 2012)	ml/min	802.616	76.618	1.01	20.4
eGFR_PANEL_ (CKD-EPI 2021)	ml/min	803.010	76.224	1.03	20.3
eGFR_COMB_ (EKFC)	ml/min	806.657	72.577	1.14	21.5
eGFR_CYSC_ (CKD-EPI 2012)	ml/min	812.841	66.393	0.878	22.4
eGFR_CYSC_ (EKFC)	ml/min	814.916	64.318	1.01	22.9
eGFR_CR_ (CKD-EPI 2021)	ml/min	815.541	63.693	1.12	24.1
eGFR_CR_ (CKD-EPI 2009)	ml/min	815.593	63.641	1.08	24.0
eGFR_PANEL_ (CKD-EPI 2021)	ml/min per 1.73 m^2^	816.234	63.000	1.05	23.7
eGFR_CYSC_ (EKFC)	ml/min per 1.73 m^2^	816.990	62.244	1.12	23.8
eGFR_CR_ (EKFC)	ml/min	817.281	61.953	1.10	24.4
eGFR_COMB_ (CKD-EPI 2021)	ml/min per 1.73 m^2^	818.417	60.817	1.00	24.3
eGFR_CYSC_ (CKD-EPI 2012)	ml/min per 1.73 m^2^	818.785	60.449	0.924	24.3
eGFR_COMB_ (CKD-EPI 2012)	ml/min per 1.73 m^2^	819.495	59.739	0.993	24.6
eGFR_COMB_ (EKFC)	ml/min per 1.73 m^2^	820.993	58.241	1.15	25.1
eCrCL (Cockcroft-Gault)	ml/min per 1.73 m^2^	823.588	55.646	0.854	25.3
eCrCL (Cockcroft-Gault)	ml/min	829.684	49.550	0.727	26.9
eGFR_CR_ (EKFC)	ml/min per 1.73 m^2^	835.764	43.470	1.01	29.3
eGFR_CR_ (CKD-EPI 2021)	ml/min per 1.73 m^2^	836.399	42.835	1.01	29.5
eGFR_CR_ (CKD-EPI 2009)	ml/min per 1.73 m^2^	836.433	42.801	0.967	29.5

CKD–EPI, chronic Kidney Disease Epidemiology Collaboration; COMB, combined creatinine and cystatin C; CR, creatinine; CV, coefficient of variation; CYSC, cystatin C; eCrCL, estimated creatinine clearance; eGFR, estimated glomerular filtration rate; EKFC, European Kidney Function Consortium; IIV_CL_, interindividual variability on gentamicin clearance; mGFR, measured glomerular filtration rate; PANEL, filtration marker panel (creatinine, cystatin C, β–2 microglobulin, and β–trace protein).

**Figure 2:**
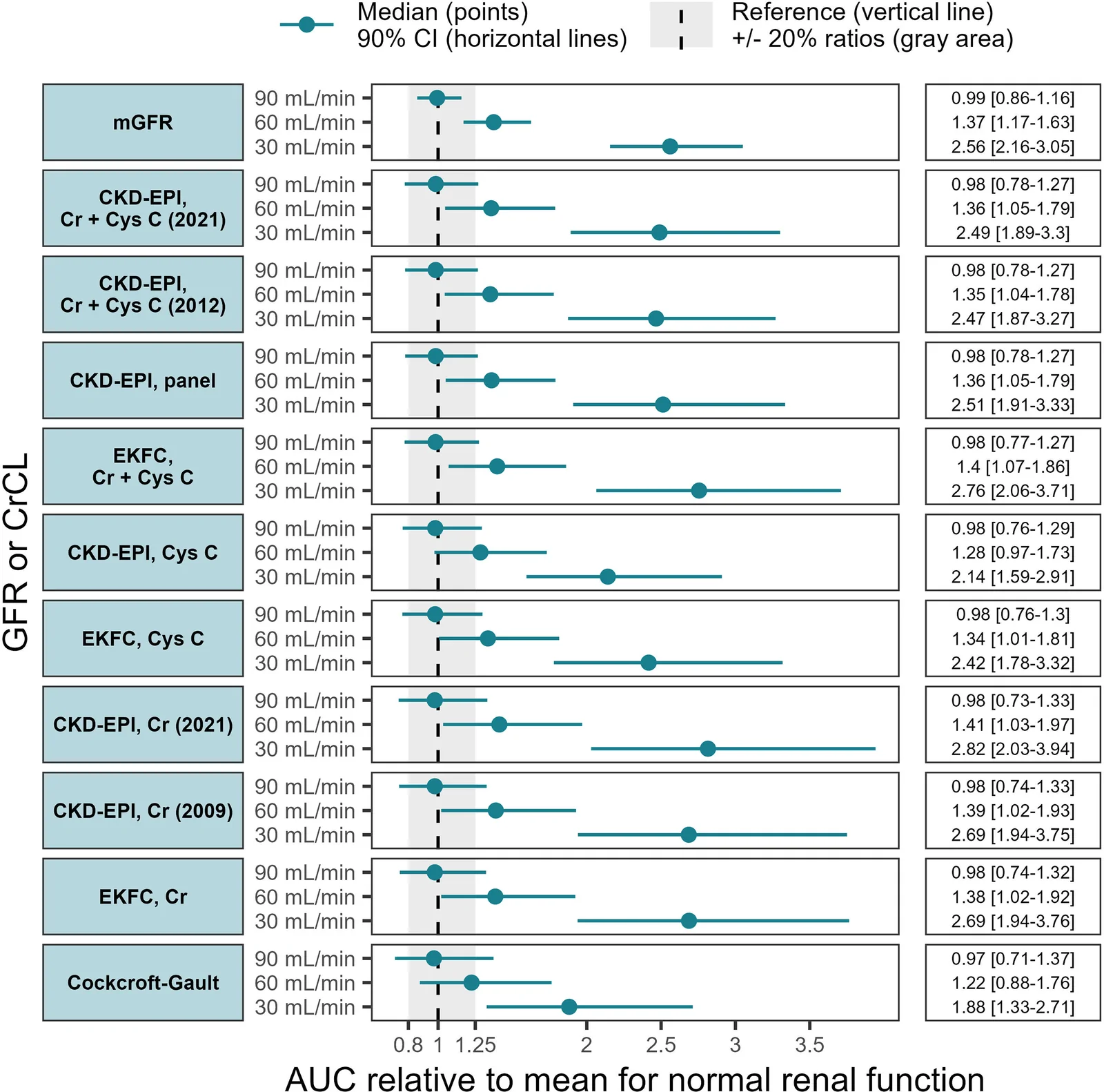
Forest plot showing the impact of different kidney function measures as covariates on gentamicin clearance in population pharmacokinetic models. Simulated gentamicin exposure was generated using 11 covariate models for subjects with mGFR, eGFR, or eCrCL values of 90, 60, and 30 ml/min. The simulated dose was 350 mg, and 1000 simulations were run for each subject. A GFR or CrCL of 90 ml/min was considered to be normal kidney function and the mean AUC was used as reference (ratio of 1, dashed line). Median AUC (points) and 90% CI (horizontal lines) are shown as ratios relative to the reference for each covariate and subject group. Boxes on the right state median [90% CI]. CKD–EPI, Chronic Kidney Disease Epidemiology Collaboration; Cr, creatinine; Cys C, cystatin C; EKFC, European Kidney Function Consortium; mGFR, mGFR, measured glomerular filtration rate.; panel, biomarker panel consisting of creatinine, cystatin C, β-trace protein and β-2 microglobulin.

Model-based simulations of AUC for subjects with mGFR, eGFR or eCrCL of 90, 60 and 30 ml/min relative to the mean AUC for normal kidney function (using 90 ml/min as the reference) showed that mGFR, eGFR_COMB_, and eGFR_PANEL_ used as covariates on clearance provided greater accuracy and precision compared with other approaches (Figure 2). For mGFR, the 90% CIs of the 3 groups were narrower than of the other group and did not overlap, and although there was a small overlap between the CI for 60 ml/min and the reference area of the 0.8–1.25 bioequivalence criterion. eCrCL showed overlapping CIs between the 90 and 30 ml/min categories, and the CI for 60 ml/min intersected the reference line. The remaining eGFR equations showed overlapping 90% CIs for sequential categories but not for the lowest and highest categories. Although the CIs were generally wider for low kidney function than for normal kidney function, they spanned 1.5 units or more for eGFR_CR_ and eGFR_COMB_ from the CKD-EPI and EKFC equations. The performance metrics of GFR equations relative to mGFR (i.e., bias, relative bias, root mean squared error, P20, and P30) showed trends similar to the results of the covariate analysis (Table 4).

**Table 4:** Performance of various eGFR equations compared with mGFR, all in absolute units (ml/min) (sorted from largest to smallest P30)

Equation	Bias (95% CI)	Relative bias (95% CI)	P30 (95% CI)	P20 (95% CI)	RMSE (95% CI)
eGFR_COMB_ (CKD-EPI 2012)	−0.22 (−1.6, 2.7)	−0.48 (−2.6, 4.0)	96.2 (90.4, 100)	86.5 (76.9, 94.2)	0.137 (0.103, 0.172)
eGFR_COMB_ (EKFC)	−0.85 (−2.9, 0.93)	−1.1 (−6.3, 1.7)	96.2 (90.4, 100)	88.5 (78.8, 96.2)	0.150 (0.109, 0.189)
eGFR_PANEL_ (CKD-EPI 2021)	0.98 (−0.024, 3.9)	2.5 (−0.051, 7.6)	96.2 (90.4, 100)	86.5 (76.9, 94.2)	0.149 (0.109, 0.188)
eGFR_COMB_ (CKD-EPI 2021)	3.7 (1.5, 5.9)	6.3 (2.9, 11.3)	94.2 (86.5, 100)	80.8 (69.2, 90.4)	0.152 (0.114, 0.192)
eGFR_CR_ (EKFC)	2.9 (−0.60, 5.7)	6.3 (-0.97, 10.2)	92.3 (84.6, 98.1)	75.0 (63.5, 86.5)	0.190 (0.142, 0.241)
eGFR_CYSC_ (EKFC)	−6.4 (−8.1, −2.4)	−9.0 (−14, -4.8)	86.5 (76.9, 94.2)	75.0 (63.5, 86.5)	0.198 (0.158, 0.233)
eGFR_CYSC_ (CKD-EPI 2012)	−8.6 (−10.1, −4.8)	−13.9 (−18.3, −9.6)	80.8 (69.2, 90.4)	67.3 (53.8, 78.8)	0.231 (0.190, 0.268)
eCrCL (Cockcroft-Gault)	−3.6 (−6.6, 0.015)	−6.3 (−12.5, −0.39)	80.8 (69.2, 90.4)	59.6 (46.2, 73.1)	0.266 (0.210, 0.322)
eGFR_CR_ (CKD-EPI 2009)	9.9 (6.0, 12.5)	16.9 (10.3, 23.7)	75.0 (63.5, 86.5)	53.8 (40.4, 67.3)	0.245 (0.190, 0.301)
eGFR_CR_ (CKD-EPI 2021)	13.5 (9.7, 16.6)	25.6 (15.4, 33.2)	57.7 (44.2, 71.2)	40.4 (26.9, 53.8)	0.290 (0.236, 0.346)

CI, confidence interval; CKD–EPI, Chronic Kidney Disease Epidemiology Collaboration; CR, creatinine; CYSC, cystatin C; COMB, combined creatinine and cystatin C; eCrCL, estimated creatinine clearance; eGFR, estimated glomerular filtration rate; EKFC, European Kidney Function Consortium; mGFR, measured glomerular filtration rate; P30, percentage of results for eGFR within 30% deviation from mGFR; P20, percentage of results for eGFR within 20% deviation from mGFR; PANEL, filtration marker panel (creatinine, cystatin C, β–2 microglobulin, and β–trace protein); RMSE, root mean square error.

### Final Model

After implementing mGFR as a covariate on clearance and testing the remaining potential covariates, body weight was found to be a significant covariate on the central volume of distribution (*P* < 0.01, ΔOFV = –19.93). The best model for gentamicin on the basis of these data was, therefore, a 2-compartment model with a combined proportional and additive error model, IIV on all fixed effect parameters, mGFR as a covariate on clearance and body weight as a covariate on the central volume of distribution. Model-estimated pharmacokinetic parameters are shown in Table 2, and bootstrap results (on the basis of 99.8% successful runs) are shown in Supplementary Table S5. Goodness-of-fit plots and visual predictive checks are shown in Supplementary Figures S6–S8.

## Discussion

This proof-of-concept study included 52 older medical patients with poor appetite and a high prevalence of chronic disease, polypharmacy, malnutrition, low muscle mass, and chronic inflammation. We developed a popPK model for gentamicin administered through i.v. as a representative renal-risk medication that is exclusively eliminated by glomerular filtration. Nine eGFR equations, 1 eCrCL equation, and mGFR were analyzed as covariates for gentamicin clearance. Among these, mGFR expressed in ml/min yielded the highest accuracy for predicting gentamicin clearance. Among the eGFR equations, eGFR_COMB,_ and eGFR_PANEL_ expressed in ml/min performed best, whereas eGFR_CR_ expressed in ml/min per 1.73 m^2^ performed worst. Regardless of the equation, expressing GFR in ml/min rather than ml/min per 1.73 m^2^ provided substantially better prediction of gentamicin clearance.

Few other studies have used a population pharmacokinetic modeling approach for comparative assessment of eGFR equations on drug pharmacokinetics in high-risk patients, but none of these studies included mGFR.^36^^,^^37^^,^^61^ To our knowledge, this is the first study to include mGFR as a covariate in this type of model-based comparative analysis, enabling comparison of both eGFR and mGFR as covariates on gentamicin clearance. In line with our expectations, we found that mGFR using a gold standard method was associated with the greatest improvement in model fit for gentamicin data.

Although the earlier studies focused on critically ill adults, the medical patients in this study were older (median 79.5 vs. 61–68 years) with lower BMI (median 23 vs. 25–30 kg/m^2^) and lower eGFR (median 67 vs. 80–96 mL/min based on eGFR_CR_ from CKD-EPI 2009).^36^^,^^37^^,^^61^ Similar to the previous studies, we observed greater improvements in model fit with eGFR_COMB_ compared with eGFR_CR_ or eCrCL and for absolute units (ml/min) compared with BSA-indexed units (ml/min per 1.73 m^2^). As drug clearance is expressed in nonindexed flow units (l/h), this was not unexpected. However, comparison of eGFR_COMB_ with mGFR indicates that there remains substantial room for improvement in eGFR equations in this high-risk group of older medical patients with poor appetite.

The performance of eGFR and eCrCL equations compared with mGFR in older adults has been investigated previously.^34^^,^^62^^,^^63^ Among older medical patients, Iversen *et al.*^34^ found that eGFR_COMB_ performed better than eGFR_CR_ or eGFR_CYSC_—particularly among patients with malnutrition—whereas eGFR_PANEL_ yielded only minimal improvement. Similarly, Potok *et al.*^62^ found that both eGFR_CR_ and eGFR_CYSC_ (from CKD-EPI 2009 and 2012, respectively) were outperformed by GFR_COMB_ from CKD-EPI 2012, with P30 (95% CI) values (compared with iohexol plasma-based mGFR) of 79% (76%–82%), 86% (84%–89%), and 91% (88%–93%), respectively. These values align with those found in the present study (76% [64%–87%], 81% [69%–90%], and 96.2% [90%–100%], respectively; Table 4), indicating overall better performance of eGFR_COMB_ compared with single-biomarker equations.

The simulations of gentamicin exposure presented in Figure 2 suggest that using creatinine-only equations, particularly eCrCL, may be problematic for dose adjustment of renal-risk medications in older medical patients. Although the simulations for mGFR clearly show a clinically relevant impact on gentamicin exposure, the overlapping 90% CIs between categories and the reference line for eCrCL indicate that the data does not allow for a definite conclusion regarding the impact and clinical relevance of the covariate. In clinical settings and in model-informed drug development, using poorly performing equations carries risks of either overdosing, causing adverse effects, or underdosing, leading to an insufficient intended effect. With eGFR_COMB_, eGFR would reflect mGFR more accurately, as seen for the median AUCs in this study, providing a more reliable basis for dose adjustment. Although the wider 90% CIs indicate less precise estimates of AUC using eGFR_COMB_ as a covariate compared with mGFR, they are narrower and overlap less than those for the models on the basis of single-biomarker equations. Furthermore, eGFR_COMB_ are more feasible in a clinical setting than conducting a full GFR measurement.

There remains ongoing debate about the best method for estimating renal clearance during drug pharmacokinetic studies. Current guidelines from the European Medicines Agency and the U.S. Food and Drug Administration on evaluation of the pharmacokinetics of drugs in patients with impaired kidney function accept the use of any eGFR or eCrCL equation, as long as it is widely accepted and clinically applicable.^64^^,^^65^ Both sets of guidelines mention drawbacks of eCrCL but do not require the use of more accurate methods. Thus, eCrCL continues to be used for drug development despite strong evidence for the inaccuracy of this method compared with both mGFR and actual drug clearance. Similarly, both sets of guidelines recommend using absolute units but still allow the use of BSA-indexed units. As such, nothing precludes drug manufacturers from publishing dosing recommendations in BSA-indexed units despite the limitations. The findings of this study are particularly relevant for older medical patients with poor appetite who are prescribed renal-risk medications that are dosed according to eCrCL or eGFR_CR_. These vulnerable patients frequently receive drugs, such as rivaroxaban, morphine, gabapentin, metformin, or even chemotherapeutic agents, for which dosing errors may have severe consequences.^34^^,^^66^, ^67^, ^68^, ^69^ It is uncertain to what extent possible discrepancies between the method used during drug development and that used in the clinic postapproval may impact exposure. Thus, this issue may require more evidence from specific as well as more general populations. As such, it is important for regulatory agencies, industry, and hospitals to carefully consider the choice of equation for estimating kidney function. Altogether, our results support increased use of eGFR_COMB_ in older medical patients with poor appetite, as well as increased use of mGFR when precise GFR determination is necessary, in line with the most recent recommendations from the KDIGO 2024 guidelines.^18^

The main strength of this study was the use of mGFR, which enabled the popPK-based evaluation of mGFR, eGFR, and eCrCL in relation to a model drug for renal clearance. Another strength of the study was the focus on older medical patients with poor appetite (on the basis of clinically applicable inclusion criteria), who are often underrepresented in clinical trials despite being overrepresented in acute care settings. Finally, the popPK model of gentamicin performed well, and the structure and parameter estimates were generally consistent with previously developed gentamicin popPK models in similar patient populations.^51^^,^^70^, ^71^, ^72^ Implementing mGFR as a covariate on clearance reduced IIV from 46.4% to 13.8% (12.3% in the final model with body weight as a covariate on volume) and substantially improved model fit, confirming the suitability of gentamicin as a renally excreted model drug for this type of analysis. The focus of this study has been to represent a relatively homogeneous and vulnerable study population, in which low appetite represents a comparable clinical phenomenon among patients with a BMI within or below the normal range. An important limitation of the study is the focus on a specific patient population at a single study site, which may limit the generalizability of the results to other study populations, for example, overweight older individuals or those receiving different types of medication.

In this proof-of-concept study of older medical patients with poor appetite, mGFR was associated with the greatest improvement in popPK model fit for gentamicin. Among the eGFR equations, eGFR_COMB_ showed the greatest improvement, whereas eGFR_PANEL_ did not provide additional improvement over eGFR_COMB_. Altogether, our findings strongly support the use of eGFR_COMB_ expressed in absolute units (ml/min) to optimize dosing of renally excreted medications in older medical patients with poor appetite.

## Disclosure

The authors declare no conflicts of interest.

## Supplementary Material

Supplementary File (PDF) **Figure S1.** Flowchart. **Figure S2.** Schematic representation of the structure of the gentamicin base model. **Figure S3.** Goodness–of–fit plots for the gentamicin base model. **Figure S4.** Observed and model–predicted gentamicin plasma concentrations on ordinary and logarithmic scale. **Figure S5.** Visual predictive check of the base model. **Figure S6.** Goodness–of–fit plots for the final gentamicin model. **Figure S7.** Observed and model–predicted gentamicin plasma concentrations on ordinary and logarithmic scale. **Figure S8.** Visual predictive check of the final model. **Table S1.** Selection criteria. **Table S2.** Equations for estimation GFR. **Table S3.** Detailed reasons for patient inclusion and exclusion. **Table S4.** Detailed reasons for dropout after inclusion. **Table S5.** Model estimates and bootstrap results for the base and final models of gentamicin.

## Footnotes

Supplemenary Material
Supplementary File (PDF)
**Figure S1.** Flowchart. **Figure S2.** Schematic representation of the structure of the gentamicin base model. **Figure S3.** Goodness–of–fit plots for the gentamicin base model. **Figure S4.** Observed and model–predicted gentamicin plasma concentrations on ordinary and logarithmic scale. **Figure S5.** Visual predictive check of the base model. **Figure S6.** Goodness–of–fit plots for the final gentamicin model. **Figure S7.** Observed and model–predicted gentamicin plasma concentrations on ordinary and logarithmic scale. **Figure S8.** Visual predictive check of the final model. **Table S1.** Selection criteria. **Table S2.** Equations for estimation GFR. **Table S3.** Detailed reasons for patient inclusion and exclusion. **Table S4.** Detailed reasons for dropout after inclusion. **Table S5.** Model estimates and bootstrap results for the base and final models of gentamicin.
