# Supplementary material for: Impact of mGFR Versus eGFR on the Pharmacokinetics of a Renally Excreted Model Drug in Older Medical Patients: A Proof-of-Concept Trial
Source: Kidney Int Rep. 2026 Aug 17;11(11):107020. doi: 10.1016/j.ekir.2026.107020 (PMC13599624; doi:10.1016/j.ekir.2026.107020)
Supplement: Supplementary file 1 [file mmc1.pdf]

Supplementary materials

Impact of mGFR *verus* eGFR on the pharmacokinetics of a renally excreted model drug in older medical patients: a proof-of-concept trial

Ida K Storgaard*, Morten B Houlind*, Louise WS Christensen, Rikke L Nielsen, Aino L Andersen, Olivia Bornæs, Helle G Juul-Larsen, Johnny KH Dang, Baker N Jawad, Izzet Altintas, Juliette Tavenier, Esteban Porrini, Esben Iversen, Morten Damgaard, Ove Andersen, Mads Hornum, Trine M Lund

* Shared first authorship

Correspondence: Morten Baltzer Houlind (morten.baltzer.houlind@regionh.dk)

Figure S1. Flowchart. **Table S1.** Selection criteria. **Table S2.** Equations for estimation GFR.**Table S3.** Detailed reasons for patient inclusion and exclusion. **Table S4.** Detailed reasons for dropout after inclusion. **Figure S2**. Schematic representation of the structure of the gentamicin base model. **Table S5.** Model estimates and bootstrap results for the base and final models of gentamicin. **Figure S3**. Goodness–of–fit plots for the gentamicin base model. **Figure S4.** Observed and model–predicted gentamicin plasma concentrations on ordinary and logarithmic scale. **Figure S5.** Visual predictive check of the base model. **Figure S6.** Goodness–of–fit plots for the final gentamicin model. **Figure S7** Observed and model–predicted gentamicin plasma concentrations on ordinary and logarithmic scale. **Figure S8** Visual predictive check of the final model.

Flowchart and detailed reasons for patient inclusion and exclusion

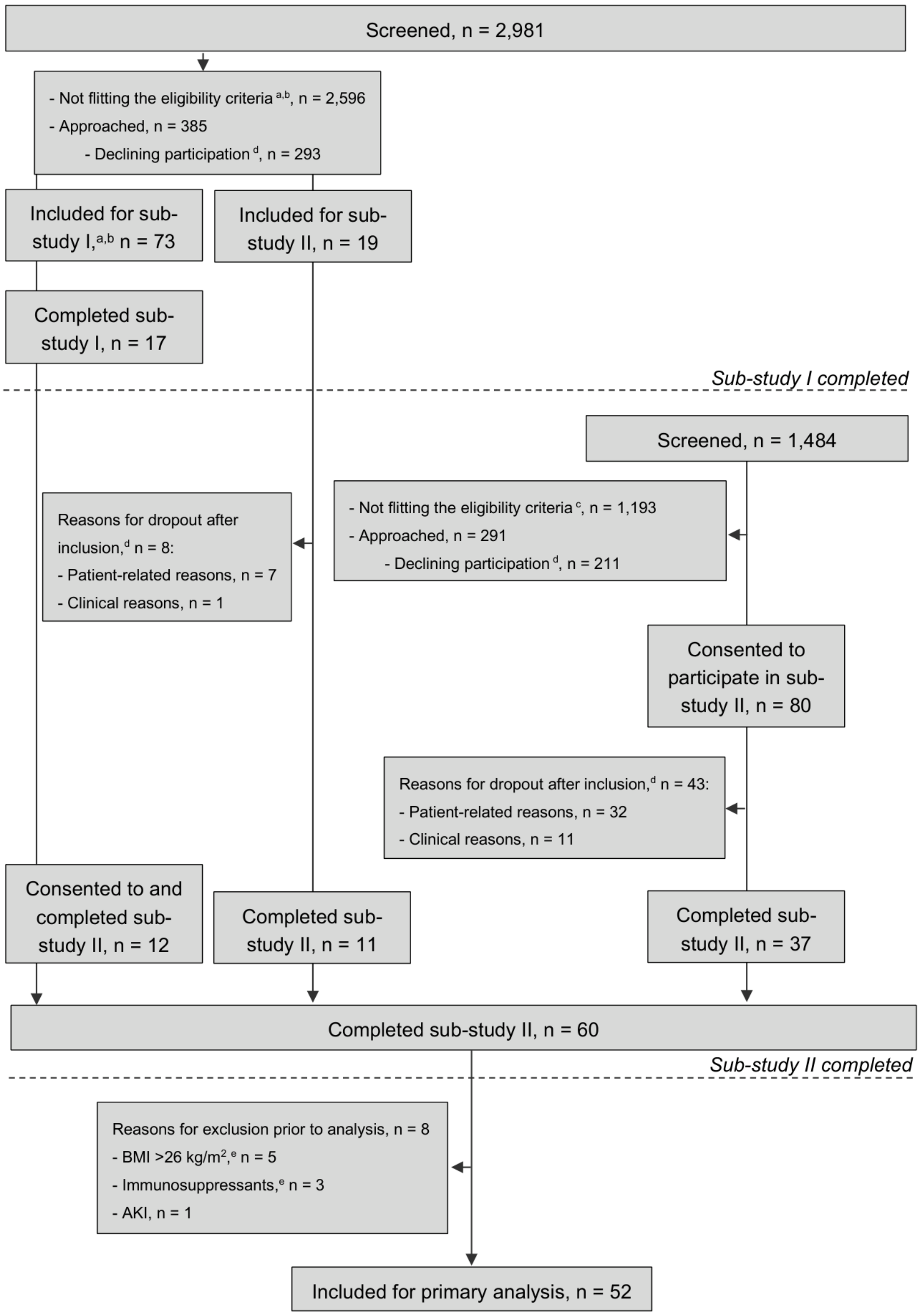

Figure S1 Flowchart of patient inclusion in the study. The present study is sub-study II.
[a,b]See references for details on inclusion and exclusion applied in sub-study I. Patients who consented to participate in sub-study I were also invited to participate in sub-study II.
[c]Reasons for exclusion prior to inclusion are detailed in **Table S2**.
[d]Reasons for dropout after inclusion are detailed **Table S3** – including the 17 patients that were included during the period of sub-study I.
[e]One patient had both BMI >26 kg/m^2 and was using an immunosuppressant.
Abbreviations: AKI, acute kidney injury; BMI, body mass index; n, number of patients.

Selection criteria

Table S1. Selection criteria. For all equations, S_{Cr} is in mg/dL, S_{CysC}, S_{B2M} and S_{BTP} are in mg/L. eGFR is calculated in body surface area-indexed units (mL/min/1.73 m^2) for all equations except eCrCL (Cockcroft-Gault), where it is in absolute units (mL/min).

Inclusion criteria:

- age ≥65 years
- the ability to read, understand, and speak Danish
- the ability to cooperate cognitively and physically (e.g., absence of dementia, unconsciousness, or severe immobility)
- BMI <30 kg/m^2
- postmenopausal status, defined as a history of at least 12 months without menstruation
- a Simplified Nutritional Appetite Questionnaire (SNAQ) score ≤14 points

Exclusion criteria:

- severe personality disorder
- significant psychiatric disorder beyond mild to moderate depression
- allergy to gentamicin
- terminal illness
- active cancer treatment or disseminated cancer
- known brain or kidney tumor
- history of liver transplantation
- eGFR ≤15 mL/min/1.73 m^2 for more than three months (kidney failure) or dialysis treatment
- high risk of nephrotoxicity due to existing pharmacotherapy
- ascites
- significant edema on trial days
- isolation due to infection precautions

Patients who completed sub-study II were eligible for inclusion in the present study based on the following criteria:

- BMI ≤26 kg/m^2
- No treatment with immunosuppressants on the trial day or within the preceding 7 days
- No presence of acute kidney injury on the trial day

A pragmatic tolerance interval between BMI 25 and 26 was applied to account for minor weight fluctuations between inclusion and the trial day, thereby avoiding unnecessary exclusions without compromising cohort characteristics.

Equations for estimating GFR

Table S2 eGFR equations. For all equations, S_{Cr} is in mg/dL, S_{CysC}, S_{B2M} and S_{BTP} are in mg/L. eGFR is calculated in body surface area-indexed units (mL/min/1.73 m^2) for all equations except eCrCL (Cockcroft-Gault), where it is in absolute units (mL/min).

Abbreviation and reference		Equation
**Single: Creatinine**		
eCrCL (Cockcroft-Gault) [S1]	=	$\frac{(140-\mathrm{AGE})\times \mathrm{WT}}{72\times \mathrm{S_{Cr}}} \times 0.85$ [if female]
$eGFR_{CR}$ (CKD-EPI 2009) [S2]	=	$\mathrm{A_1} \times (\mathrm{S_{Cr}}/\mathrm{B})^{-\mathrm{C_1}} \times 0.9929^{\mathrm{AGE}} \times 1.159$ [if Black]<br>A_1: 144 if female, 141 if male<br>B: 0.7 if female, 0.9 if male<br>C_1: 0.329 if female and $S_{Cr} \leq 0.7$, 0.411 if male and $S_{Cr} \leq 0.9$, 1.209 if $S_{Cr} > B$
$eGFR_{CR}$ (CKD-EPI 2021) [31]	=	$\mathrm{A_2} \times (\mathrm{S_{Cr}}/\mathrm{B})^{-\mathrm{C_2}} \times 0.9938^{\mathrm{AGE}}$<br>$A_2$: 143 if female, 142 if male<br>B: 0.7 if female, 0.9 if male<br>C_2: 0.241 if female and $S_{Cr} \leq 0.7$, 0.302 if male and $S_{Cr} \leq 0.9$, 1.200 if $S_{Cr} > B$
$eGFR_{CR}$ (EKFC) [S3]	=	$107.3 \times (\mathrm{S_{Cr}}/\mathrm{Q})^{-\alpha} \times 0.990^{\mathrm{AGE}-40}$ [if AGE > 40]<br>α: 0.322 if $S_{Cr}/Q < 1$, 1.132 if $S_{Cr}/Q \geq 1$<br>Q: median S_{Cr} for reference population
**Single: Cystatin C**		
$eGFR_{CYSC}$ (CKD-EPI 2012) [S4]	=	$133 \times (\mathrm{S_{CysC}}/0.8)^{-\beta_1} \times 0.9962^{\mathrm{AGE}} \times 0.932$ [if female]<br>β_1: 0.499 if $S_{CysC} \leq 0.8$, 1.328 if $S_{CysC} > 0.8$
$eGFR_{CYSC}$ (EKFC) [S5]	=	$107.3 \times (\mathrm{S_{CysC}}/\mathrm{Q})^{-\alpha} \times 0.990^{\mathrm{AGE}-40}$ [if AGE > 40]<br>α: 0.322 if $S_{CysC}/Q < 1$, 1.132 if $S_{CysC}/Q \geq 1$<br>Q: median S_{CysC} for reference population
**Combination: Creatinine + Cystatin C**		
$eGFR_{COMB}$ (CKD-EPI 2012) [S4]	=	$\mathrm{A_3} \times (\mathrm{S_{Cr}}/\mathrm{B})^{-\mathrm{C_3}} \times (\mathrm{S_{CysC}}/0.8)^{-\beta_2} \times 0.9952^{\mathrm{AGE}}$<br>$A_3$: 130 if female, 135 if male<br>B: 0.7 if female, 0.9 if male<br>C_3: 0.248 if female + $S_{Cr} \leq 0.7$, 0.207 if male + $S_{Cr} \leq 0.9$, 0.601 if $S_{Cr} > B$<br>β_2: 0.375 if $S_{CysC} \leq 0.8$, 0.711 if $S_{CysC} > 0.8$
$eGFR_{COMB}$ (CKD-EPI 2021) [31]	=	$\mathrm{A_3} \times (\mathrm{S_{Cr}}/\mathrm{B})^{-\mathrm{C_4}} \times (\mathrm{S_{CysC}}/0.8)^{-\beta_3} \times 0.9952^{\mathrm{AGE}}$<br>$A_3$: 130 if female, 135 if male<br>B: 0.7 if female, 0.9 if male<br>C_4: 0.219 if female + $S_{Cr} \leq 0.7$, 0.144 if male + $S_{Cr} \leq 0.9$, 0.544 if $S_{Cr} > B$<br>β_3: 0.323 if $S_{CysC} \leq 0.8$, 0.778 if $S_{CysC} > 0.8$
$eGFR_{COMB}$ (EKFC) [S5]	=	Median of $EKFC_{Cr}$ and $EKFC_{CysC}$
**Panel: Creatinine, Cystatin C, β-2 Microglobulin, β-Trace Protein**		
$eGFR_{PANEL}$ (CKD-EPI 2020) [S6]	=	$\mathrm{A_4} \times (\mathrm{S_{Cr}}/\mathrm{B})^{-\mathrm{C_5}} \times (\mathrm{S_{CysC}}/0.8)^{-\mathrm{D}} \times (S_{B2M})^{-0.103} \times (\mathrm{S_{BTP}}/0.6)^{-\mathrm{E}} \times 0.996^{\mathrm{AGE}}$<br>$A_4$: 123 if female, 131 if male<br>B: 0.7 if female, 0.9 if male<br>C_5: 0.243 if female + $S_{Cr} \leq 0.7$, 0.295 if male + $S_{Cr} \leq 0.9$, 0.471 if $S_{Cr} > B$<br>D: 0.519 if $S_{CysC} \leq 0.8$, 0.423 if $S_{CysC} > 0.8$<br>E: 0.004 if $S_{BTP} \leq 0.6$, 0.177 if $S_{BTP} > 0.6$

Abbreviations: B2M, β–2–microglobulin; BTP, β–trace protein; CKD–EPI, Chronic Kidney Disease Epidemiology Collaboration; CR, creatinine; CYSC, cystatin C; COMB, combined creatinine and cystatin C; eCrCL, estimated creatinine clearance; eGFR, estimated glomerular filtration rate; EKFC, European Kidney Function Consortium; PANEL, filtration marker panel (creatinine, cystatin C, β-2 microglobulin, β-trace protein); S_X, serum concentration of X.

Supplementary References:

S1. Cockcroft DW, Gault MH. Prediction of creatinine clearance from serum creatinine. Nephron. 1976;16(1):31–41.

S2. Levey AS, et al. A New Equation to Estimate Glomerular Filtration Rate. Annals of Internal Medicine. 2009;150(9):604–12.

S3. Pottel H, et al. Development and Validation of a Modified Full Age Spectrum Creatinine–Based Equation to Estimate Glomerular Filtration Rate: A Cross–sectional Analysis of Pooled Data. Ann Intern Med. 2021;174(2):183–91.

S4. Inker LA, et al. Estimating glomerular filtration rate from serum creatinine and cystatin C. N Engl J Med. 2012;367(1):20-29. doi:10.1056/NEJMoa1114248.

S5. Pottel H, et al. Cystatin C–Based Equation to Estimate GFR without the Inclusion of Race and Sex. N Engl J Med. 2023;388(4):333–43.

S6. Inker LA, et al. A New Panel–Estimated GFR, Including β_2–Microglobulin and β–Trace Protein and Not Including Race, Developed in a Diverse Population. Am J Kidney Dis. 2021;77(5):673–83 e1

Table S3. Detailed reasons for exclusion prior to inclusion in sub-study II. In total, 1,484 were screened exclusively for sub-study II, 1,193 did not meet the eligibility criteria, and 291 were approached, of whom 211 declined participation.

Reason	n (%)
**Not fulfilling eligibility criteria (n= 1,193)**	
Not fulfilling inclusion criteria (screen failure, SNAQ or BMI)	420 (28.3)
Other reasons	202 (14.4)
Not available in patient room	169 (12.0)
Isolation due to infection precaution	159 (11.3)
Active cancer treatment or disseminated cancer	132 (9.4)
Terminal illness	30 (2.1)
Significant psychiatric disorder beyond mild to moderate depression	29 (2.1)
Chronic eGFR* ≤15 mL/min/1.73 m^2 at the time of inclusion	25 (1.8)
Known tumor in the brain or kidneys	11 (0.8)
Observation for COVID-19	7 (0.5)
Significant edema on trial days	6 (0.4)
Ascites	1 (0.1)
Hepatic transplant	1 (0.1)
Allergy to the active ingredient gentamicin	1 (0.1)
**Declined participation although fulfilling eligibility criteria (n=211)**	
No interest in the study	109 (7.8)
Too tired to participate	87 (6.2)
No reason given	8 (0.6)
Study too demanding	6 (0.4)
Participating in another research study	1 (0.1)

Abbreviations: AMI, acute myocardial infarction; BMI, body mass index; eGFR, estimated glomerular filtration rate; EMR, electronic medical record; n, number of patients; NYHA, New York heart association; SNAQ, simplified nutritional appetite questionnaire.

* Calculated by the creatinine-based Chronic Kidney Disease Epidemiology equation (2009) without race factor.

Table S4 Detailed reasons for dropout after inclusion, n = 51.

Reasons, n (%)	n = 8	n = 43
Too tired to participate	5 (26.3)	14 (32.6)
Unknown reasons	0 (0.0)	13 (30.2)
Death	1 (5.3)	5 (11.6)
No longer fulfilling the eligibility criteria	0 (0.0)	2 (4.7)
Do not have time	1 (5.3)	5 (11.6)
Illness	0 (0.0)	4 (9.3)
No interest in the study	1 (5.3)	0 (0.0)

Abbreviation: n, number of patients.

Schematic structure of the gentamicin base model

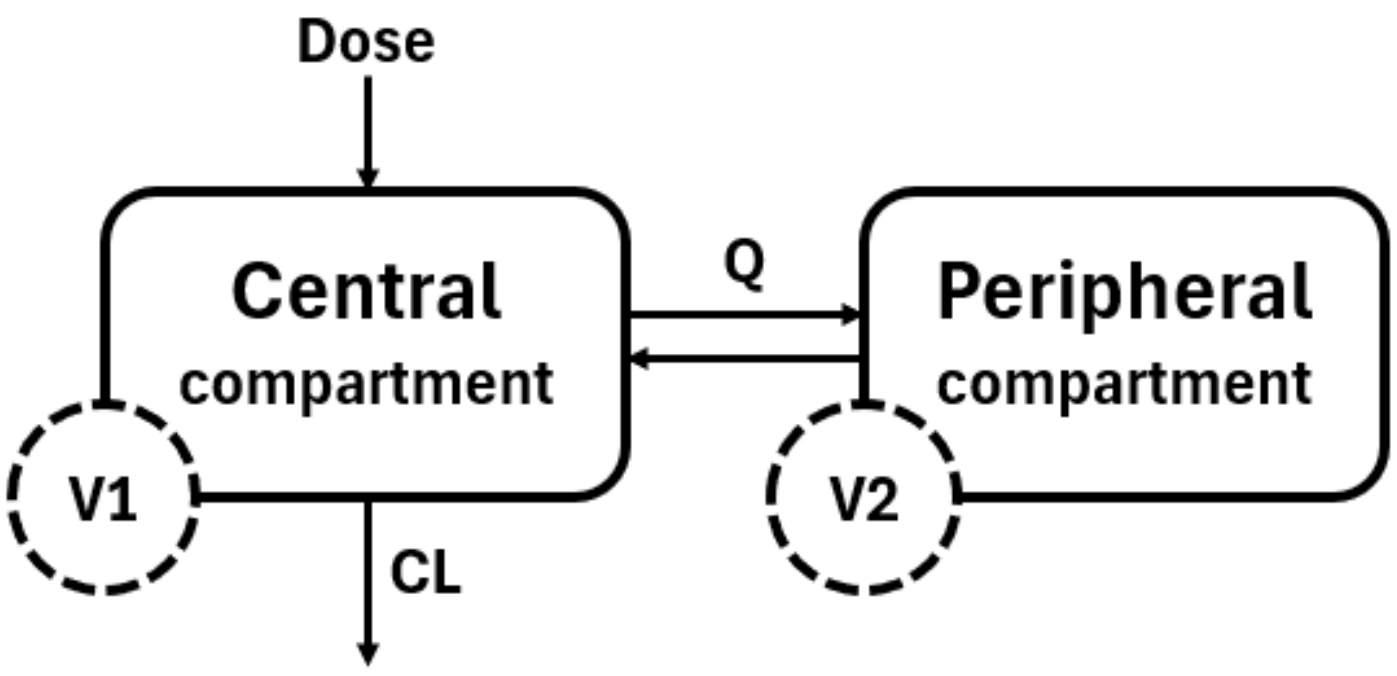

Figure S2 Schematic representation of the structure of the gentamicin base model.
Abbreviations: CL, elimination clearance; Q, inter-compartmental clearance; V1 and V2, the distribution volumes of the central and peripheral compartments, respectively.

Bootstrap results for the base and final models

Table S5 Model estimates and bootstrap results for the base and final models of gentamicin.

		Base model		*Final model*	
**Parameter**	**Description**	**Estimate (RSE)**	**Bootstrap mean (95% CI)**	**Estimate (RSE)**	**Bootstrap mean (95% CI)**
**CL**	Elimination clearance (L h^{-1})	2.27 (6.3%)	2.25 (1.99-2.55)	2.31 (2.0%)	2.30 (2.20-2.42)
**Q**	Intercompartmental clearance (L h^{-1})	7.55 (17.2%)	7.26 (4.65-10.5)	8.05 (16.5%)	7.74 (5.31-10.8)
**V1**	Distribution volume of the central compartment (L)	8.95 (6.7%)	8.79 (6.94-11.0)	9.69 (4.8%)	9.74 (8.62-10.8)
**V2**	Distribution volume of the peripheral compartment (L)	7.62 (7.8%)	7.59 (6.59-8.66)	7.70 (7.6%)	7.62 (6.66-8.73)
$\theta_{CL\text{-}mGFR}$	Covariate effect of mGFR on clearance (power function)	–	–	1.06 (7.9%)	1.05 (0.892-1.22)
$\theta_{V1\text{-}WT}$	Covariate effect of body weight on V1 (power function)	–	–	1.39 (38%)	1.42 (0.317-2.47)
IIV_{CL}	Interindividual variability on CL (%, [shrinkage])	46.4 (22.5%) [1%]	45.9 (17.9-65.6)	12.3 (19.0%) [10%]	12.2 (6.06-16.4)
IIV_{Q}	Interindividual variability on Q (%, [shrinkage])	55.6 (18.8%) [24%]	61.5 (0-85.7)	38.5 (43.1%) [37%]	44.2 (0-70.1)
IIV_{V1}	Interindividual variability on V1 (%, [shrinkage])	60.6 (30.7%) [0%]	69.4 (0-116)	44.8 (35.3%) [2%]	45.8 (0-71.0)
IIV_{V2}	Interindividual variability on V2 (%, [shrinkage])	27.3 (21.1%) [21%]	28.3 (7.61-38.4)	24.7 (23.1%) [23%]	25.1 (7.93-34.5)
RUV_{CV}	Proportional residual error	0.0806 (9.7%)	0.0779 (0.0651-0.0961)	0.0847 (8.5%)	0.0822 (0.0687-0.101)
RUV_{SD}	Additive residual error (mg L^{-1})	0.215 (18.7%)	0.193 (0.0533-0.377)	0.238 (18.8%)	0.229 (0.0970-0.379)

Abbreviations: CI, confidence interval; CV, coefficient of variance; IIV, inter-individual variability; RSE, relative standard error.

Goodness–of–fit plots and visual predictive check for the gentamicin base model

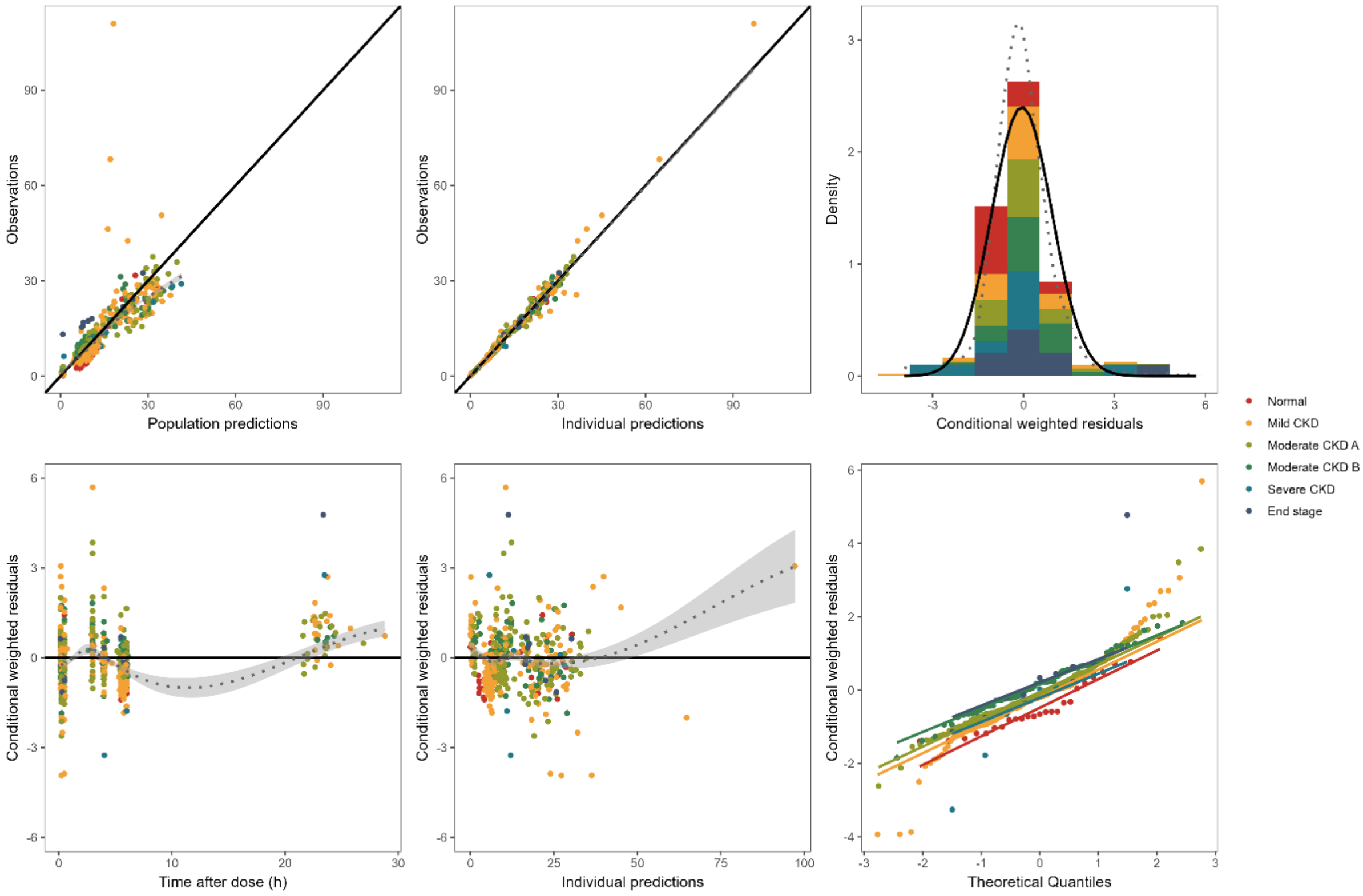

Figure S3 Goodness–of–fit plots for the gentamicin base model.

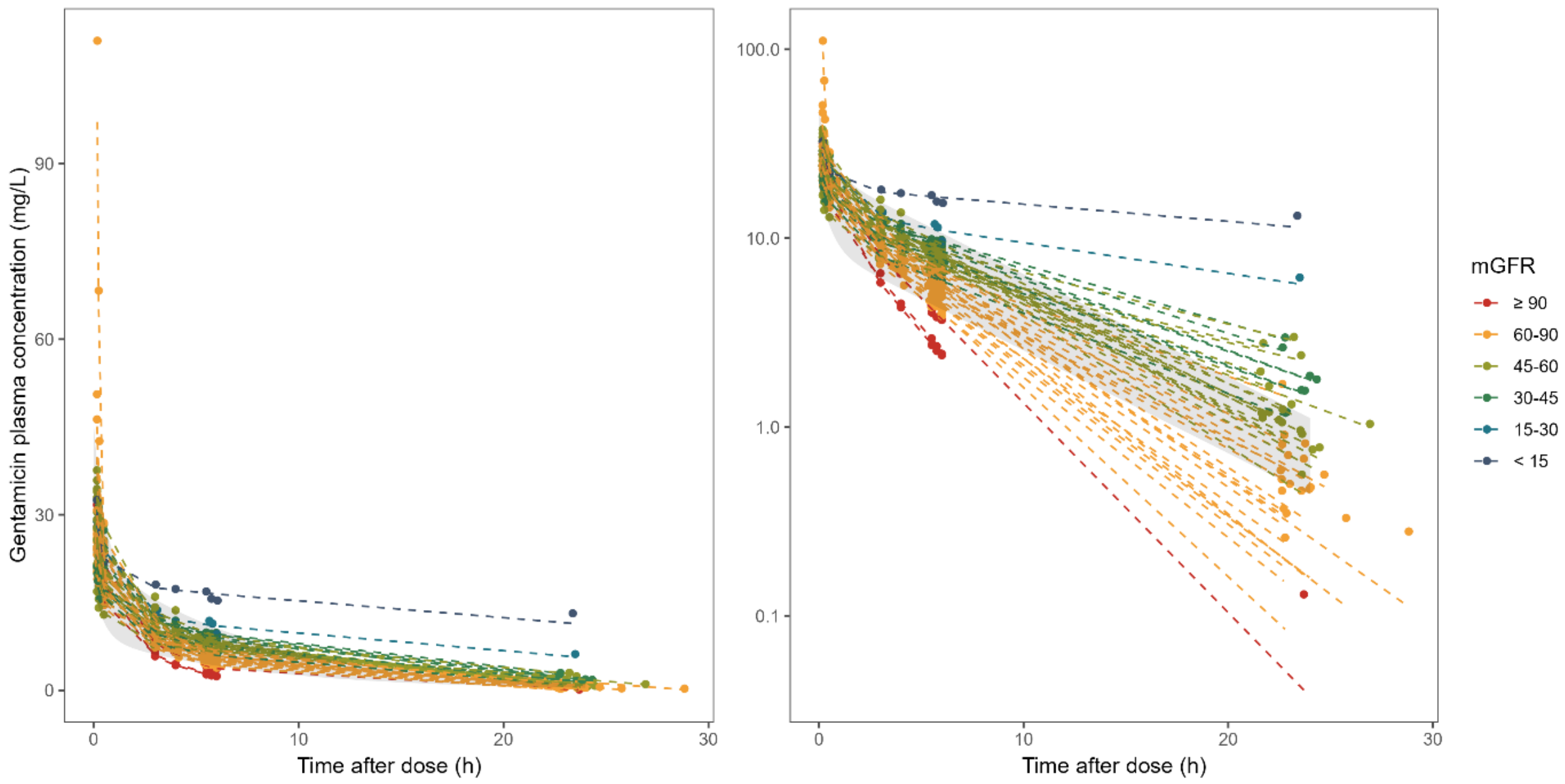

Figure S4 Observed and model–predicted gentamicin plasma concentrations on ordinary and logarithmic scale. Points are observations, dashed lines are individual predictions, and the shaded area is population predictions for the weight–based doses (5 mg/kg) administered in this study, simulated with the base model (not adjusted for any covariates). Points and dashed lines are colored according to the individual's measured glomerular filtration rate (mGFR, mL/min/1.73 m^2).

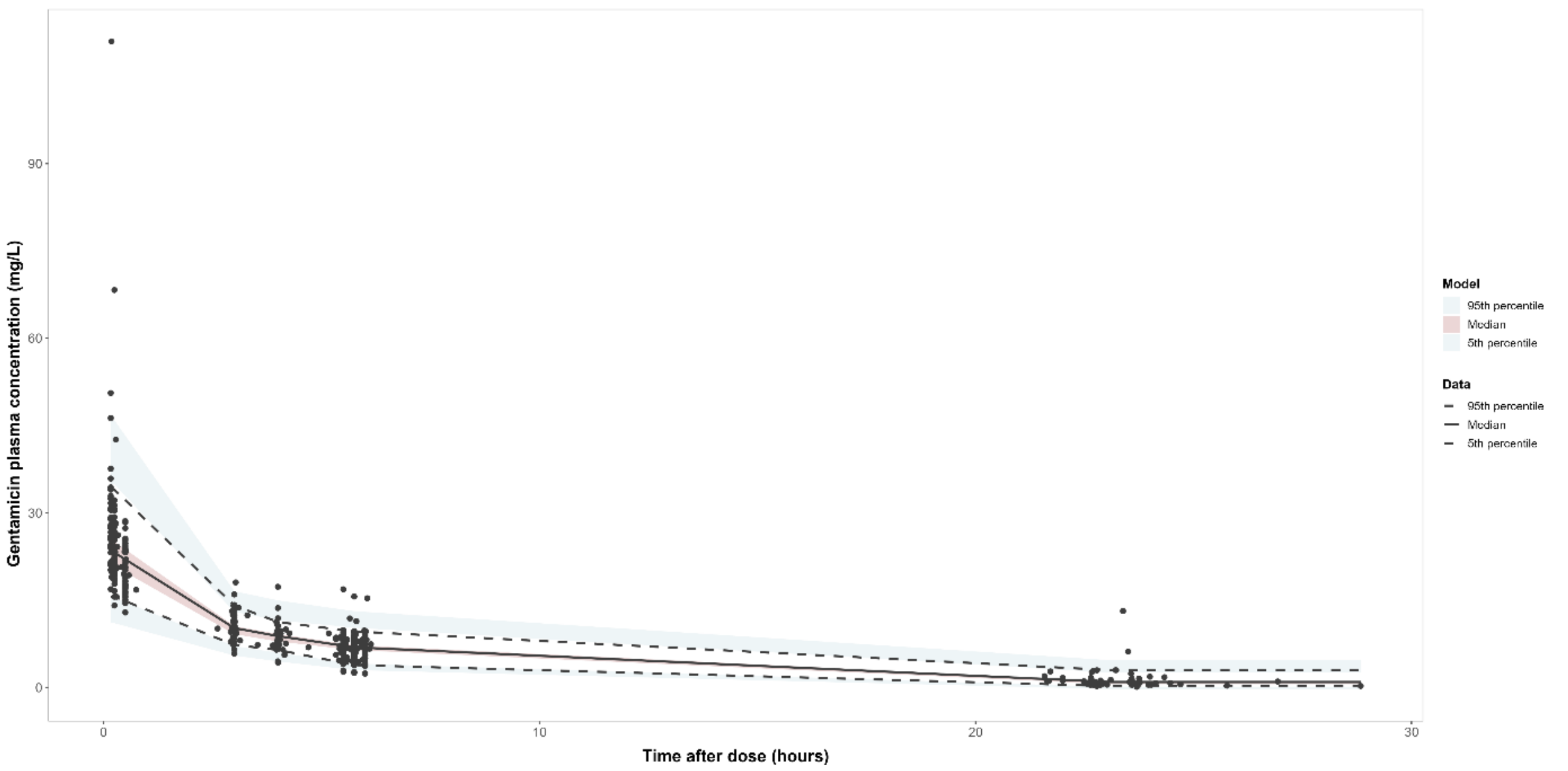

Figure S5 Visual predictive check of the base model (n = 1000).

Goodness–of–fit plots and visual predictive check for the final gentamicin model

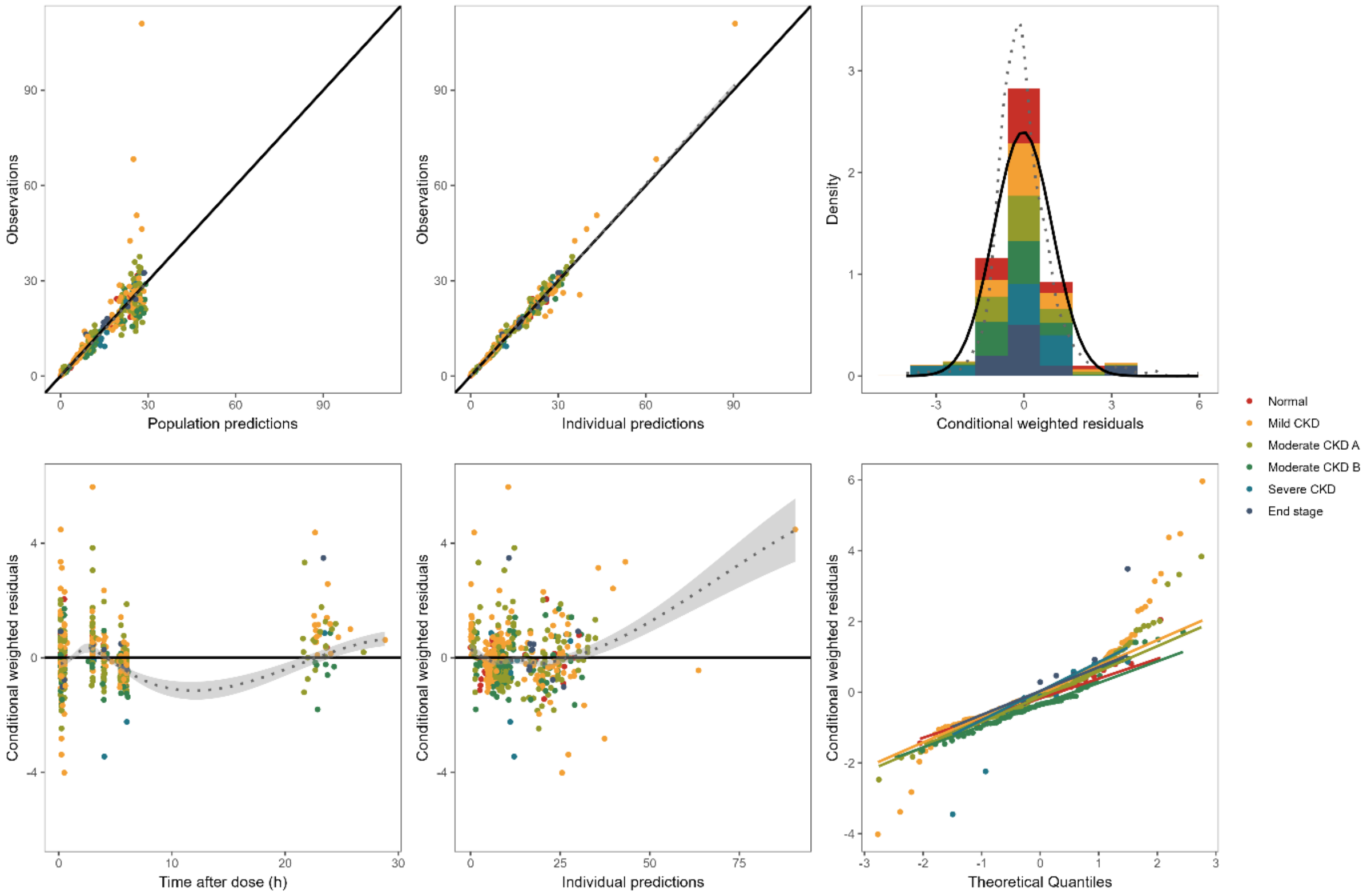

Figure S6 Goodness–of–fit plots for the final gentamicin model with mGFR as a covariate on clearance and weight as a covariate on the volume of the central compartment.

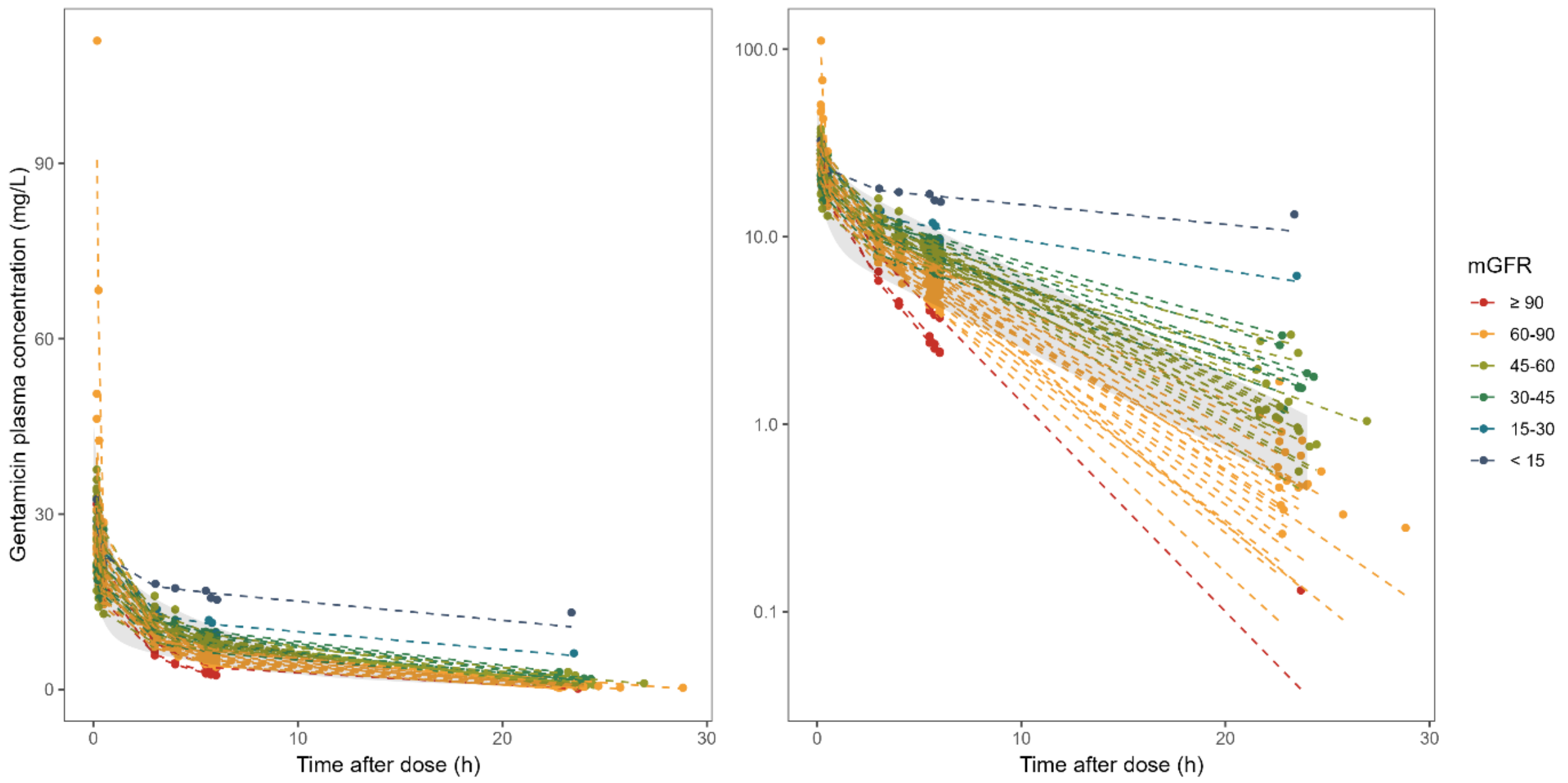

Figure S7 Observed and model–predicted gentamicin plasma concentrations on ordinary and logarithmic scale. Points are observations, dashed lines are individual predictions, and the shaded area is population predictions for the weight–based doses (5 mg/kg) administered in this study, simulated with the final model (with mGFR as a covariate on clearance and weight as a covariate on the volume of the central compartment). Points and dashed lines are colored according to the individual's mGFR (mL/min/1.73 m^2).

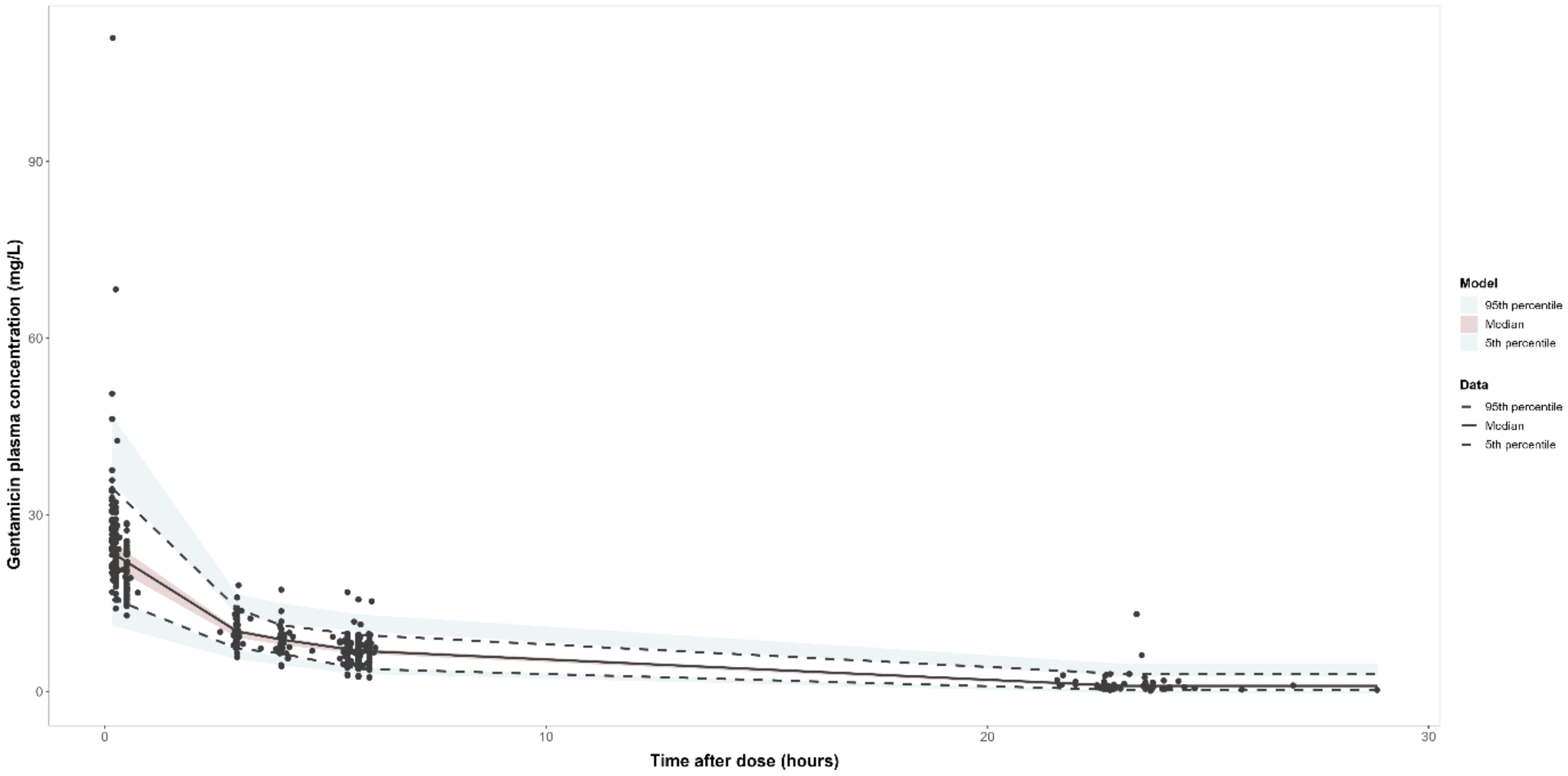

Figure S8 Visual predictive check of the final model (n = 1000).
